# The Crosstalk between Gut Microbiota and Nervous System: A Bidirectional Interaction between Microorganisms and Metabolome

**DOI:** 10.3390/ijms241210322

**Published:** 2023-06-19

**Authors:** Monica Montagnani, Lucrezia Bottalico, Maria Assunta Potenza, Ioannis Alexandros Charitos, Skender Topi, Marica Colella, Luigi Santacroce

**Affiliations:** 1 Department of Precision and Regenerative Medicine and Ionian Area-Section of Pharmacology, School of Medicine, University of Bari “Aldo Moro”, Policlinico University Hospital of Bari, Piazza G. Cesare 11, 70124 Bari, Italy; monica.montagnani@uniba.it (M.M.); mariaassunta.potenza@uniba.it (M.A.P.); 2 School of Technical Medical Sciences, “Alexander Xhuvani” University of Elbasan, 3001-3006 Elbasan, Albania; bottalico.lu@gmail.com (L.B.); skender.topi@uniel.edu.al (S.T.); 3 Pneumology and Respiratory Rehabilitation Division, Maugeri Clinical Scientific Research Institutes (IRCCS), 70124 Bari, Italy; 4 Interdisciplinary Department of Medicine, Microbiology and Virology Unit, School of Medicine, University of Bari “Aldo Moro”, Piazza G. Cesare, 11, 70124 Bari, Italy; marycolella98@gmail.com (M.C.); luigi.santacroce@uniba.it (L.S.)

**Keywords:** human microbiota, metabolome, immunity, biochemistry, gut/brain axis, neurotransmitters, Enteric Nervous System (ENS), dysbiosis, probiotics, psychobiotics

## Abstract

Several studies have shown that the gut microbiota influences behavior and, in turn, changes in the immune system associated with symptoms of depression or anxiety disorder may be mirrored by corresponding changes in the gut microbiota. Although the composition/function of the intestinal microbiota appears to affect the central nervous system (CNS) activities through multiple mechanisms, accurate epidemiological evidence that clearly explains the connection between the CNS pathology and the intestinal dysbiosis is not yet available. The enteric nervous system (ENS) is a separate branch of the autonomic nervous system (ANS) and the largest part of the peripheral nervous system (PNS). It is composed of a vast and complex network of neurons which communicate via several neuromodulators and neurotransmitters, like those found in the CNS. Interestingly, despite its tight connections to both the PNS and ANS, the ENS is also capable of some independent activities. This concept, together with the suggested role played by intestinal microorganisms and the metabolome in the onset and progression of CNS neurological (neurodegenerative, autoimmune) and psychopathological (depression, anxiety disorders, autism) diseases, explains the large number of investigations exploring the functional role and the physiopathological implications of the gut microbiota/brain axis.

## 1. Introduction

### 1.1. The Instestinal Metaboloma’s Concept

**Figure 1:**
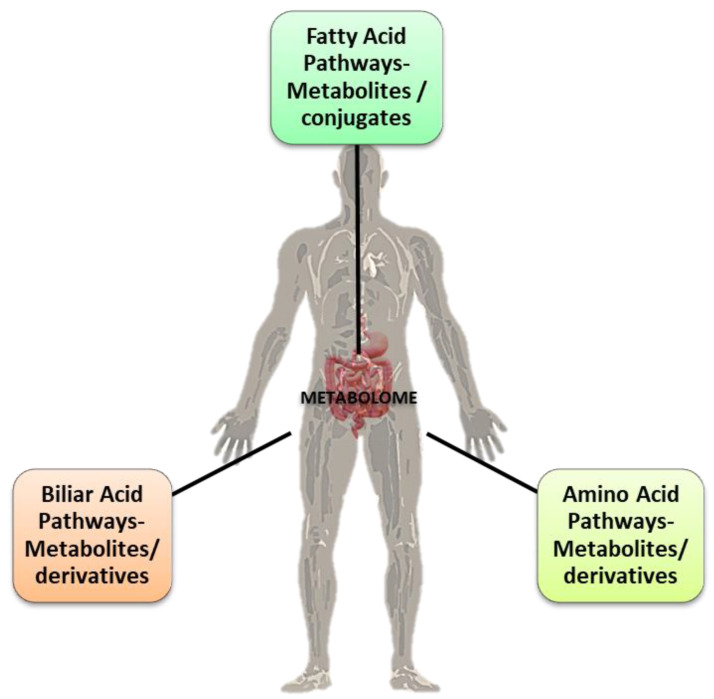
The three main pathways of metabolites’ (conjugates and derivates) formation in the gut via the microbiota. Some of them are correlated with the gut microbiota eubiosis (interspecies healthy balance) and the “good” balance for the normal physiological homeostasis functions of the host’s organism. Credits: Original figure by I.A. Charitos.

The microbiota is a complex, interconnected bio-system of microorganisms in the human body whose activities vary according to the interaction between the microbial components and the different organs of the host. The widest microorganism population, the gut microbiota, is connected to various cross-talking microbial axes such as the gut/lung, gut/brain, gut/skin, and bladder/gut/brain axes [1]. Since ancient times, physicians such as Hippocrates and, later, Galen recognized that “*maldigestion is the root of all suffering*”, noting that “*all diseases originate in the intestine*” and taking advantage of available treatments such as herbal infusions to smooth diseases symptoms [2,3]. The microorganisms inhabiting our gut exceed 10^14^, with our microbiota possessing 100 times more genes than our own genome, and a total weight of approximately 1–2 kg (close to the weight of our brain) [4,5,6]. In light of this background, the emerging field of metabolomics may expand our current knowledge on diseases pathophysiology and help to develop a “personalized therapy” for each patient [7]. Metabolomics is the systematic study of the unique chemical “fingerprints” left by specific cellular processes which involve metabolites, small molecules, and intermediate and/or end products of cellular metabolism, including those derived from the intestinal microbiota (Figure 1) [8]. 

One major challenge of systems biology and functional genomics is to integrate genomic, transcriptomic, proteomic, and metabolomic information to attain a better understanding of cell biology. In the context of metabolomics, a metabolite is generally defined as any molecule of low molecular weight (<1.5 kDa) [9], with some exceptions depending on the sample and detection methods; for example, macromolecules such as lipoproteins and albumin can be reliably detected using Nuclear Magnetic Resonance (NMR) spectroscopy in blood plasma [10]. In metabolomics, it is customary to refer to “primary” and “secondary” metabolites: while a primary metabolite participates directly in normal growth, development, and reproduction, the secondary metabolite is not directly involved in these processes, but usually has an important ecological function. Nevertheless, in metabolomic analyses performed on human samples, it is more common to describe metabolites as endogenous (produced by the host organism) or exogenous [11]. The metabolome, which includes the totality of all low molecular weight (1 < kDa) endogenous compounds in a biological sample, represents the metabolic state of an organism under certain conditions. Metabolites and their levels reflect the phenotype (influenced by its genetic background and environmental organization) of a biological system at a given moment in time [12,13]. Nowadays, research and study of the metabolome contributes to clinical and pharmaceutical research, as they provide the possibility of identifying biochemical changes associated with specific diseases to understand, in depth, the mechanism of action of new substances, as well as indicate biomarkers for the early diagnosis of various diseases. As a paradigmatic condition, the metabolic syndrome (MetS or syndrome X) reflects a cluster of metabolic abnormalities associated with cardiovascular disease and type II diabetes mellitus. For this, as for several other diseases which share a metabolic and cardiovascular pathogenesis, understanding the relationships and interactions between the genetic background, microbiota composition, and metabolome profile may help to plan personalized and more effective treatments [2,5]. 

### 1.2. Metabolome Analysis Techniques

Up until the 1990s, germs and the intestinal microbiota were studied by common methods including cultures media and microscopy. Besides the costs and the time of procedures, the main disadvantage of these was that the totality of the microbial populations colonizing our organism could not be satisfactorily discovered. Today, the applied techniques rely on DNA isolation and amplification of the 16S ribosomal RNA (rRNA) gene, present in all bacteria, archaea, and fungi and not significantly changed during evolution. These techniques, combined with polymerase chain reaction (PCR) and metagenomic sequencing, are effective in characterizing various microbial strains [14,15], allowing to discover new bacterial species via post-genomic genome reconstruction [15].

For each metabolomic study, the most appropriate analysis method depends on the objectives, and usually a compromise between sensitivity, selectivity, and speed is chosen. Considering the extremely large number of potential metabolites in each metabolome, which include lipids, amino acids, peptides, volatile alcohols, organic acids, vitamins, and complex natural products, it is implicit that their analysis is a difficult challenge. For this reason, a single analytical test is not sufficient to detect all of the metabolites found in a sample [16]. NMR and mass spectrometry MS are among the main analytical techniques applied in metabolomic studies. Each method has advantages and disadvantages, and they can be applied in addition to each other. 

MS is a highly sensitive method for detecting and quantifying, but also determining the structure of, a compound with a single measurement [17]. This technique contributes significantly to the visualization of the metabolic profile, providing broad coverage in metabolite detection. However, a significant number of compounds in complex mixtures give variable responses during MS, making the interpretation of results difficult; in addition, MS often leads to the degradation of analytes, or the disruption of their molecular complex interactions, ultimately leading to the loss of important information. Finally, MS-based metabolomics analyses are disadvantaged in terms of repeatability, inter-evaluator fidelity, and reproducibility, and thus require the use of complex statistical tools for processing the results [18]. 

NMR does not require the separation of analytes, is a non-destructive method (thus allowing for re-use of the same samples for further analysis), and provides information on the molecular structure of particularly complex compounds. The sample preparation required is simple and makes NMR useful for metabolic profiling analyses of biological fluids. However, because there are large numbers of metabolites in the range of biological fluids, their signals often overlap, making the identification and quantification of such molecules difficult [19].

Recent developments in NMR and MS, as well as their combination, promise to significantly improve both the identification and quantification of metabolites in samples and speed up the process of identifying new biomarkers. With these methods, the mass of a molecule is given by calculating the mass/charge ratio of the ion (*m*/*z*). Ions are created by the absorption or loss of charge from a neutral molecule [20], in a step-by-step procedure which finally provides the molecular weight of the compounds and the intensity of the analyzed signal, as well as structural information [21]. 

For the metabolomics analysis of biological samples, the coupling of MS with chromatography provides important advantages, ultimately resulting in more precise quantification of individual metabolites [22]. The main separation methods used in conjunction with MS are liquid chromatography (high-performance or ultra-high performance liquid chromatography, HPLC, UHPLC), gas chromatography, and capillary electrophoresis [23,24,25].

Metabolomic analyses require the detection of metabolites with high discrimination and sensitivity. For this reason, methodologies have been developed to fragment the precursor ions so that further structural information is extracted (tandem MS TANDEM MS or MS/MS). With the aid of fragmentation methodologies, an increase in the specificity and, thus, the sensitivity of MS-based quantification methodologies (monitoring of multiple fragmentation reactions; monitoring of multiple MRM reactions) is simultaneously achieved [26,27]. Various types of mass analyzers have been developed. Low resolution analyzers such as the simple quadrupole analyzer, linear ion trap, and quadrupole ion trap have a resolution of ∼1000, while high resolution analytes, such as the time of flight (time of flight, TOF) analyzer, the Fourier transform ion cyclotron resonance analyzer, and the orbital ion trap, have a resolution of 10,000–20,000,000 and are considered more suitable for detecting and identifying more metabolites. The choice of the specific mass spectrometry technique depends on the final goal of the metabolomic study and the organizational cost [28,29]. 

## 2. The Metabolic Processes of Gut Microorganisms

The structure of the small intestine has several features that support nutrient absorption while also being an ecosystem for various microorganisms. The dominant genera of bacteria are *Bacteroidota*, *Bacillota*, and *Actinomycetota*. In the colon, *Bacteroidetes* and *Lachnospiracae* (*Bacillota* phylum) are the most common bacteria [8,10]. This is partly because more acidic conditions and a higher oxygen concentration are observed in the small intestine compared to the large intestine [5,30]. However, the small intestine exhibits a microbial environment in which facultative anaerobic bacteria predominate (they are resistant to the presence of bile acids and antimicrobial agents) and can utilize the simple carbohydrates present in the small intestine environment [30,31]. A lower degree of bacterial diversity is shown in the ileum than in the colon, with many species of the phyla *Pseudomonadota* and *Clostridium* spp. being present. Gene expression analysis has shown that they are involved in metabolism and in specific cellular pathways which are dedicated, among others, to carbohydrate entry [5,6,32]. With respect to the whole body, the widest and densest composition of microbes is observed in the colon and cecum. Here, resident germs are responsible for the catabolism of undigested polysaccharides and the lack of simple carbohydrates facilitates the growth of anaerobic bacteria, thus carrying out the degradation of polysaccharides, such as the *Bacteroidaceae* and *Clostridiaceae* families. Bacteria contribute to sustaining the methyl group cycle, which in turn relies on the folate cycle to transport methyl residuals (-CH3) [32]. Various intermediates of the cycle act as co-substrates in: different biosynthetic pathways such as the purine pathways, the availability of methyl group donors, and the redox balance of the cell through trans-sulphuration. Furthermore, methyl group metabolism plays an important role in embryogenesis, stem cell maintenance, hematopoiesis, DNA and histone methylation, and immune cell function [32,33,34]. 

**Figure 2:**
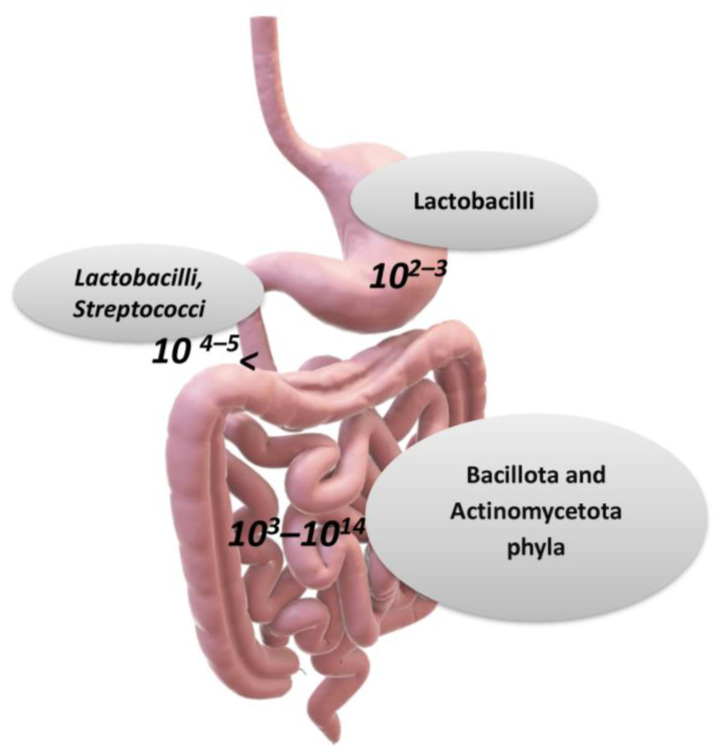
The main taxa of bacteria in the gastro-intestinal tract (over genera 500, and 10^12^–10^14^ microorganisms). The stomach carries about 10^2^–10^3^ bacteria, the duodenum 10^4^–10^5^, the ileum 10^8^–10^9^, and the bacteria and colon 10^13^–10^14^ (per gram of tissue or feces). Larger numbers of bacterial cells have been found in the large intestine, with 10^12^ bacteria (per gram of intestinal tissue), and the variety of bacteria is greater than that in the small intestine [1,6]. According to a hypothesis for the prevalence of genera in the human microbiota, there can be three main enterotypes, which are *Bacteroides* (enterotype 1), *Prevotella* (enterotype 2), and *Ruminococcus* (enterotype 3) Credits: Original figure by I.A. Charitos.

In 2011, the enterotype hypothesis (re-visited in 2018 by Costea and coworkers and still controversial) was proposed according to the hypothesis that the gut microbiota of each individual person can be classified into one of three main bacterial groups (enterotypes) depending on the numerical predominance of the genus of microbes it carries, and each group includes several subgroups. Therefore, the three main enterotypes are: *Bacteroides* (enterotype 1), *Prevotella* (enterotype 2), and *Ruminococcus* (enterotype 3) [6,35,36]. The prevalence of these genera of microbes is mainly determined by nutritional and environmental factors, and microbial populations can change over the lifespan (Figure 2) [2,35,36]. 

When the microbial population of gut microbiota experiences “difficult” coexistence (dysbiosis), this opens up favorable conditions for the development of some diseases [5]. Current studies show that the dysbiosis (changes in both qualitative and quantitative microbial composition) of the intestinal microbiota can create significant disturbances, such as inflammatory bowel diseases, eating disorders, allergies, autoimmunity diseases, and some forms of intestinal cancer [5,37,38,39]. Common causes include eating habits, alcohol abuse, chemical xenobiotics (such as intoxication by heavy metals, or exposure to bisphenol A), abuse of substances (such as cocaine, methamphetamines, and others), and the careless use of antibiotics, which are responsible for significant metabolic or inflammatory changes. For example, the short chain fatty acids (SCFAs), such as butyric and acetic acid, with their immunoregulatory effects, appear to be reduced in colon cancer patients. Similarly, microbial pyridoxine (vitamin B6) can stimulate the antitumor immune surveillance of the host [40,41], and this protection may be lost under gut microbiota dysbiosis. In these last years, there has been increasing attention focused on potential mechanisms which are able to correlate the intestinal microbiota with some neurodegenerative diseases such as Parkinson’s disease and Alzheimer’s disease [41,42,43,44,45]. The underlying hypothesis is that a change in gut bacterial populations may negatively reflect on the physiological activities of the nervous system.

## 3. The Metabolic Activity of Gut Microorganisms

The intestinal microbiota has several important functions, ranging from the synthesis of vitamins (such as Vitamins K, B_12_, B_9_) and the catabolism of biomolecules to the metabolism of bile salts and fibers [46,47,48]. The gut microbiota is similar to an independent “organ” that participates in bio-chemical transformations which are relevant for human physiology while also storing, converting, and recycling large amounts of energy [49,50,51]. 

**Figure 3:**
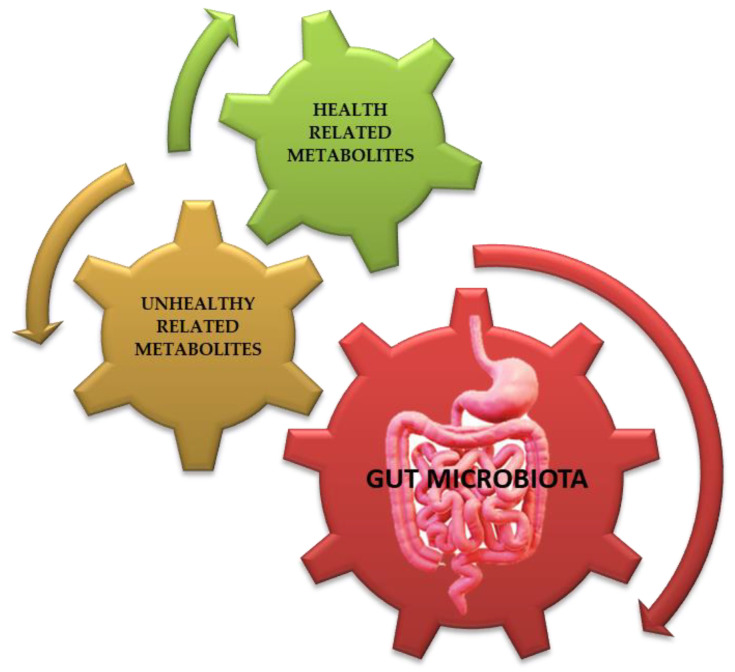
Gut microbiota’s (see red arrow) metabolites (derivates/conjugates) may be correlated with host’s health (see green arrow, such as 1,9-Nonanedicarboxylic Acid, methyl carboxylate, glycyl-L-valine, S-Carboxymethyl-L-cysteine, (Z)-3-hydroxyoctadec-11-enoic acid, 3 alpha 7 alpha-dihydroxy-5 beta-cholanic acid, and others) or those correlated with certain pathologies (see brown arrow, such as adrenic acid, arachidonic acid, cucurbit acid, carnosine, chenodeoxycholic acid-3-β-d-glucuronide, N-alfa-L-Acetyl-Arginine, N-propionyl-d-glutamine, α-Muricholic acid, and others) [53]. Credits: Original figure by I.A. Charitos.

The gastrointestinal tract, which belongs to the digestive tract, provides a place of residence and food for microorganisms while offering benefits to the host such as continuous and intense metabolic activity (aiding digestion), food absorption, mucus production, fatty acids elaboration, and the regulation of inflammatory reactions, overall contributing to the homeostasis of the immune system (such as the regular development of cytokines). The gut microbiota’s metabolites may be correlated with the host’s health (such as methyl carboxylate, glycyl-L-valine, 3 alpha, 7 alpha-dihydroxy-5 beta-cholanic acid, and others) or with certain pathologies (such as adrenic acid, carnosine, chenodeoxycholic acid-3-β-d-glucuronide, and others) (Figure 3) [2,32,52,53].

Intestinal microorganisms actively contribute to the catabolism of indigestible fibers, sugars, fats, amino acids, bile acids, and cholesterol, but also bacterial waste and endogenous mucus. The proximal part of the duodenum (between the pylorus and major duodenal papilla) contains strictly anaerobic bacteria that survive under hypoxic conditions, but also facultative anaerobic organisms that survive with and without oxygen. The most populous of the three phyla, previously mentioned, is *Bacteroidota*, which are quite versatile in their environment and, due to their high adaptability to different pH values and ability to digest both proteins and carbohydrates, can inhabit different parts of the gastrointestinal tract [1,5]. These strains help digest food to produce beneficial metabolites for the host and remove toxic byproducts from the body. The genus *Bacteroides* makes up 25% of bacteria and can, under certain conditions, show pathogenic behavior. Some *Bacteroides* can use different substances depending on their availability, due to the existence of many genes which are involved in starch metabolism. The host organism lacks the appropriate enzymes to degrade complex polysaccharides [54]. An example is *B. thetaiotaomicron*, which produces different enzymes when it senses carbohydrates in the intestinal lumen. This species is involved in the metabolism of different types of carbohydrates: dietary (β-glucans, fructans) and carbohydrates derived from the host organism. For this reason, a diet which is low in processed carbohydrates causes the production of enzymes responsible for their digestion within the mucous membrane. The major dietary carbohydrates that are a source of nutrients for *B. thetaiotaomicron* are glycans and fructans [55]. These symbiotic bacteria produce various enzymes which are responsible for the degradation of these polysaccharides, which are not metabolized by the host organism; for example, endolevanase (an enzyme produced by *B. thetaoimicron*) breaks down a type of fructan, levan (β-2, polymer 6-linked fructose). The enzymes produced by these bacteria suppress the host’s defense mechanisms and, thus, the host is unable to prevent the bacteria from digesting the glucans. However, a pilot study with 15 patients demonstrated that the oral administration of β-glucans did not influence cytokine production, and neither did the antimicrobial activity of leukocytes. β-glucans have the potential to induce immune responses by stimulating the expression of pro-inflammatory cytokines by immune cells [56]. That is, they bind to Toll-like TLR receptor pattern recognition (PRR) receptors, initiating a pathway that is involved in the activation, mainly, of macrophages and dendritic cells. When they bind to the Dectin-1 receptor, it stimulates the phosphorylation of tyrosine which is bound to the cytoplasmic tail of the receptor and initiates a signal transduction cascade that is involved in the production and release of cytokines, ROS, and chemokines, as well as in the activation of phagocytosis [57]. Also, if fructans and glucans are not broken down, indigestion occurs, as the body cannot metabolize them. Furthermore, several studies show that many people with gastrointestinal problems ascribed to gluten, have, instead, an intolerance to glucans and fructans, which cannot be metabolized when this microorganism is lacking. In a pilot study investigating the role of fructans and gluten in the development of gastrointestinal problems, patients thought to have non-celiac gluten sensitivity were placed on a glucan-only diet, some on an off-only gluten diet, and the rest with virtual complement diets. The results showed that the symptoms experienced by patients who were supposed to have gluten sensitivity were due to the consumption of glu-fructans [58]. Thus, immune suppression by bacterial enzymes is a protective mechanism against host defense, which may not be related to glycan binding to TLR and Dectin-1 receptors. The host benefits of this suppression include the uptake of oligosaccharides to meet the body’s energy needs, suppression of the inflammatory response, immune tolerance to these microorganisms, and protection against allergic responses. The digestive adaptability of *B. thetaiotaomicron* contributes to the maintenance of intestinal homeostasis by allowing the microbiota to better respond to dietary changes without altering the intestinal microbial composition [59]. It has been noted that, in some animal studies (mice), the properties of this bacterium are involved in the development of the intestine from the earliest stages of life. When infants are breastfed, it produces enzymes in the intestine that can digest monosaccharides, oligosaccharides, and polysaccharides from milk [60]. *Bacteroides* also have a large genome, whose variable expression affects their interactions with the human host. Thus, depending on external factors, these bacteria activate specific genes that can transform them from conventional to pathogenic. If the numbers of these bacteria become too high, they can migrate to other areas and cause health problems. Their resistance to bile acids and antibiotics makes them potentially pathogenic. More generally, gram-negative bacteria enter the bloodstream and induce an inflammatory response from the immune system. *Bacillota* phyla are another Gram-positive phylum that plays an important role in intestinal metabolism. Some genera of the phylum *Bacillota* (such as *Clostridia* spp.) interact strongly with the immune system. *Clostridia* spp. are the first colonizers of the gastrointestinal tract and constitute a large percentage of the total bacteria in the intestinal tract. Breastfeeding generally promotes the colonization of *Clostridia* spp. in infants, and these populations appear from the first month after birth. *Clostridia* produce compounds that keep the colonic microbiota in eubiosis. These species protect against inflammatory responses of the gastrointestinal tract, such as colitis and colon cancer [53,61,62,63]. *Faecalibacterium prausnitzii*, which belongs to the *Clostridium* IV group, is the predominant species of *Clostridia* in the intestine and constitutes more than 5% of the total number of bacteria and increases the production of anti-inflammatory molecules. *Clostridium butyricum* is one of the first colonizers in the intestinal microbiota in its development since it appears in the intestine of the newborn shortly after birth and can produce metabolites (SCFAs) which are beneficial to human health. However, not all *Clostridium* spp. are helpful, some can cause infection and even beneficial ones can become particularly harmful in different environments with altered conditions, i.e., *Clostridioides difficile*, which is an opportunistic bacterium. In mice, one strain of *C. butyricum* has been shown to protect, whereas some others are associated with infectious diseases in newborns, such as botulism and necrotizing enterocolitis [63,64,65]. Another group of bacteria found in abundance in the gastrointestinal tract is the *Actinomycetota* phyla, with a major genus being the *Bifidobacteria*. These bacteria pass through the mother’s vaginal tract to the fetus and are also present in breast milk. *Veillonella*, that use lactic acid, and *Acidaminococcus*, that use aminoacids as an energy source, are also found in abundance in the gastrointestinal tract. 

Undoubtedly, the microbiota plays an important regulatory role between humans and the environment, and many research proposals for the human microbiota are in development of the resident bacteria. The intestinal microbiota interacts in several ways with muscle tissue during exercise. Studies conducted in athletes have shown that intense competitions significantly influence the intestinal microbiota (gut/muscle axis). Experiments in vivo (animal models) have shown that butyric acid levels increase with fast running. One genus-level difference is the wide-ranging numbers of the genus *Akkermansia*. The specific members of this genus are associated with low body weight and healthy metabolic function. Elevated levels of butyric acid also activate the expression of peptide YY (tyrosine dipeptide), which stimulates satiety and increases glucose utilization to meet muscle energy needs [66]. The intestinal microbiota also activates TLR4 and TLR 5 in muscles, via lipopolysaccharides and flagellin. When TLRs are activated, pro-inflammatory cytokines are produced in the muscle. With exercise, the activation of TLR4 and TLR5 by the microbiota is suppressed, thus improving metabolic processes such as insulin sensitivity. Finally, during exercise, in one study, it appeared that numerous circulating molecules were exclusively expressed by the gut microbiota, while some others changed their concentration [1]. For example, the production of indole-3-propionic acid (derived from tryptophan metabolism) was shown to be completely dependent on the presence of bacteria and especially *Clostridium* spp. In addition, it has been demonstrated that the different bacterial modulation of bile acid metabolism and the enteric cycle may modify dietary fat absorption and concomitant lipid accumulation in the liver of animals that have a maladapted microbial localization [63,66]. These findings support the contribution of exercise in maintaining the eubiosis of the intestinal microbiota and preventing the occurrence of microbial metabolic disorders [66]. 

## 4. The Role of the Intestinal Microbiota in the Gut/Brain Axis

### 4.1. The Bio-Molecular Pathways of ENS and Brain

**Figure 4:**
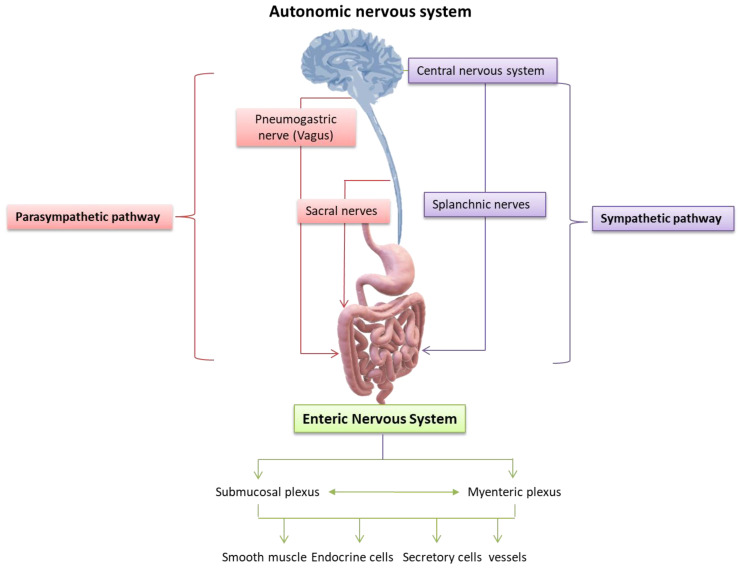
Nervous control of the gastrointestinal tract: the neural control of the gastrointestinal tract depends on the extrinsic nerves of the autonomic nervous system and the intrinsic neural networks, also known as the Enteric Nervous System (ENS). The extrinsic nerves are nerve fibers that originate outside the gastrointestinal tract and innervate its organs under the control of the autonomic (sympathetic and parasympathetic) nervous system, while regulating the activities of the neurons of the ENS. However, the ENS also functions autonomously, independently assisting the motor and secretory activities of the gastrointestinal tract. It is characteristic that even if the intestinal nerves of the autonomic system are injured or sectioned, many secretory and motor functions of the intestine are kept under the control of the ENS. Previously, the prevailing theory was that the ENS was an extension of the parasympathetic autonomic nervous system, whereas, today, there is the understanding that it constitutes an autonomic neural plexus involved in reflex and other activities of the gastrointestinal tract independently of exogenous nerve stimuli [74]. Credits: Original figure by I.A. Charitos.

The ENS interacts with the CNS in a two-way dynamic balance, which ensures the physiological activity of both systems. It is understood that any disturbance, modification, or deregulation of this interconnection can potentially affect the functionality of the other. Therefore, the interaction of the CNS with the ENS relies on the state and composition of the resident microbiota of the gastrointestinal tract. The modulatory function of the gastrointestinal tract is a complex process involving many synergistic biomechanisms. The intrinsic neural networks of the ENS are distinguished in two plexuses, the extrinsic or myenteric plexus (Auerbach’s plexus), located between the longitudinal and circular muscle layer, and the internal or submucosal plexus (Meissner’s or Remak’s plexus), located in the submucosa. The myenteric plexus is also the target of mu receptor opioids [67,68,69]. Both extend along the entire length of the body, from the esophagus to the rectum [70,71]. While the ENS can act independently from the CNS, it receives innervation from the ANS, connecting the central and enteric nervous systems. The dysfunction of this structure underlies several disorders, including Hirschsprung disease, achalasia, and gastroparesis. A unique feature of the ENS comes from its high content in neurons, which confers a relative functional autonomy to the gastrointestinal tract [71,72]. Gastroenteric motility and secretions are regulated by neuronal and hormonal control pathways based on the stimulation of specific nerve cells, which in turn activate signaling cascade responses that lead to the stimulation of intestinal smooth muscle endocrine gland cells. More specifically, the main factors involved in the regulation of gastrointestinal function are: (a) the autonomic function of the smooth muscle fibers of the gastrointestinal tract, (b) the gastrointestinal hormones, and (c) the neural control of the gastrointestinal tract (Figure 4) [72,73,74]. 

**Figure 5:**
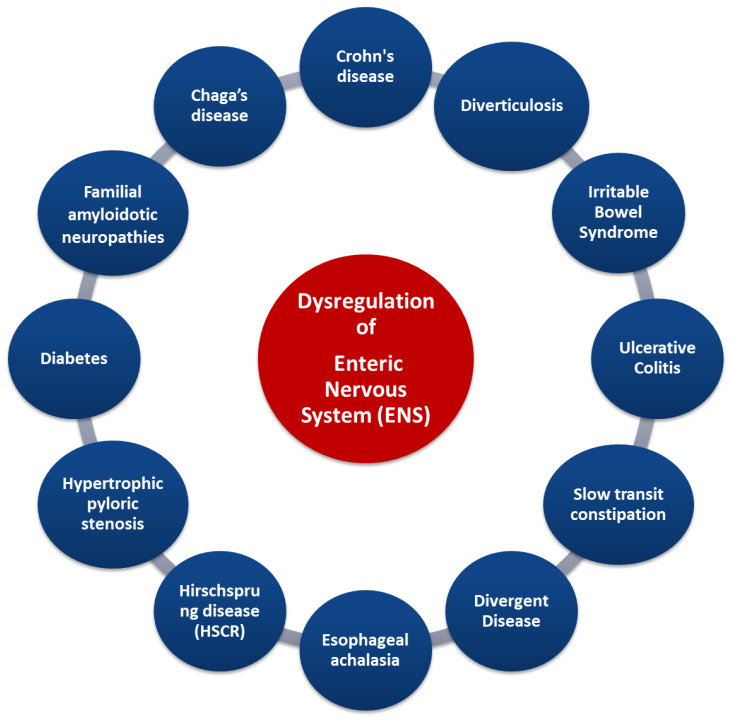
Gastrointestinal diseases associated with dysregulation of ENS. Credits: Original figure by I.A. Charitos.

The intrinsic neural networks of the ENS include neural fibers and ganglia, as well as interstitial neurons that connect afferent and efferent neurons, smooth muscle neurons, and secretory cells, forming reflex arcs within the gastrointestinal tract wall [73]. Therefore, they can coordinate the activity of the gastrointestinal tract and, without it, the effect of extrinsic nerves [74]. Previously, the prevailing perception was that the intrinsic nerves of the intestinal wall were its extension of the parasympathetic nervous system, while it is now accepted that they are an autonomic nerve plexus working independently from exogenous nerve impulses [73,74,75]. The ENS is made up of many intestinal neurons which are classified according to their location, neurochemistry, shape, length of their views, their synapses, and how they function. At present, studies on guinea pigs have led to the complete description of the functional types of intestinal neurons [76]. Therefore, the myenteric plexus mainly controls motility while the submucosal reticulum controls sweating and secretions. Reticular neurons innervate glandular cells of the mucosa and submucosa of the gastrointestinal tract, smooth muscle cells of the muscle layers, and intramural endocrine and exocrine cells. Most of the myenteric neurons are excitatory and inhibitory motor neurons that innervate the endothelial cells of smooth muscle [71,77]. These motor neurons release stimulating, or inhibitory neurotransmitters bound by smooth muscle cell receptors, regulating their function. However, sensory and interstitial neurons are also present in the myenteric plexus. Myenteric neurons control endocrine and epithelial secretion cells, including primarily sensory neurons [77]. Finally, the set of myenteric and submucosal neurons generate a multitude of intrinsic reflexes that control many motor and sensory activities in the intestinal tract. The correct cooperation of the CNS with the ENS results in a normal gastrointestinal function, while their impaired cooperation, as well as an imbalance of relevant neuro-regulatory substances, may contribute to a variety of disorders and diseases of the gastrointestinal and nutritional systems (Figure 5) [75,77,78,79].

Indeed, some diseases (such as in Crohn’s disease and irritable bowel syndrome) are characterized by an increase in the number of myenteric neurons, whereas others (such as esophageal achalasia and Hirschsprung syndrome) display a decrease in neurons. The destruction of ENS neurons due to inflammation, amyloid deposition, or other causes is related to diseases such as familial amyloid polyneuropathy [75]. Other gastrointestinal diseases (such as ulcerative colitis and obstructive ileus) are characterized by disturbances in the secretion of specific neurotransmitters. ENS deregulation can lead to the modification of gastrointestinal tract reflexes, causing a significant limitation of intestinal motility, as in patients with slow constipation [75,80,81]. The enteric neurons control the functions of the mucosa such as the electrolytic secretion and the homeostasis of the intestinal tract, while, in the intestinal epithelium, the extent to which the ENS is involved in specific enteric pathogens has been studied [76]. Importantly, understanding the interaction of the CNS and ENS via the gut/brain axis can provide not only important data for the treatment of functional disorders of the digestive system but also data about psychological disorders such as depression (even in the course of certain diseases). Several studies have now focused on the gut microbiota and how its changes are affected by stress, but have also pointed out its link with the CNS functions, as gastrointestinal bacteria activate various neural signaling pathways. The interactions between gastrointestinal microbiota and the ENS can be direct and indirect. Bacterial components can be found on the surfaces of gram-negative (LPS or polysaccharide A) or gram-positive (peptidoglycan) bacteria, and each microbial-associated molecular pattern (MAMP) is recognized by surface transmembrane Pattern Recognition Receptors (PRRs) or Toll-like endosomes (TLRs) which are expressed in myenteric neurons, enteric glial cells, and innate immune cells. Polyamines interact during stress responses, inflammation, and neuronal signalling, and short chain fatty acids involved in maintaining ENS homeostasis can activate several G protein-coupled receptors (GPCRs) and PNS, and can also inhibit the activity of histone deacetylases (HDACs). Microorganisms’ endocrinology confirms that the gut microbiota can produce an array of neurotransmitters, such as serotonin (modulates intestinal secretion and motility), dopamine, norepinephrine, and others, creating a bacteria–neuron communication in the ENS, PNS, and CNS. The commensal bacteria can stimulate the enteric glial cells (EGCs) and the mechanisms that underlie this interconnection rely on TLRs (particularly on TLR2 and TLR4). Finally, the intestinal microbiota-derived membrane vesicles facilitate the movement of signals into the intestinal microenvironment [5,82,83]. 

### 4.2. The Effect of Gut Microbiota on the CNS

In the bidirectional relationship between the gut nervous system and the CNS (the gut/brain axis), the gut microbiota plays an essential balancing role. Several possible biomechanisms through which the gut microbiota can influence the nervous system have been highlighted [84] and include, for example, the activation of the pneumogastric or vagus nerve, the production of metabolites with neuroactive properties (such as short-chain fatty acids), and the activation of the immune system [85,86,87,88]. Furthermore, bacteria synthesize many neurotransmitters and neuromodulators which are active in the nervous system, such as epinephrine, norepinephrine, serotonin, dopamine, γ-aminobutyric acid, and acetylcholine, among others. The pathways of intercellular communication networks that are sensitive to these hormones, (such as epinephrine and norepinephrine) can actively enhance the growth of Gram-negative species, such as *E. coli*, *Yersinia enterocolitica*, and *Pseudomonas aeruginosa* [89]. Norepinephrine also improves the adherence of the pathogens *E. coli* and *P. aeruginosa* to the intestinal mucosa (Table 1) [90,91]. In addition, the microbiota is particularly sensitive to small autonomic stress hormones, which are known to diffuse throughout the gastrointestinal tract.

Thus, the gut microbiota can influence the gut/brain axis through a variety of endocrine or neural pathways and immune processes [21,77].

Sensory neurons may also contribute to this dynamic balance: due to their protective role, they can perceive pathological stimuli and inform the nervous system on inflammation, temperature changes, mechanical stress, and even the presence of pathogenic biomolecules. In a series of experiments on mice pneumonia by *S. aureus*, it was shown that turning off these neurons increased the recruitment of immune system cells and the lungs’ ability to clear the bacteria, resulting in an increased survival rate. The hypothesis is that neurons limit the activity of the immune system, and that, in the case of bacterial pneumonia, the survival rate in mice is reduced. To determine how nerve cells influence the immune mechanisms, the activity of the immune system was compared in mice with lung neurons intact and in mice with lung neurons genetically or chemically disabled. Mice with non-functional neurons were able to attract more cytokines and, thus, reduce infection and bacterial shedding by producing a more rapid immune response in the early stages of infection. In contrast, mice with normal neurons showed reduced gamma- delta T cell activity. Moreover, pulmonary neurons may enable the release of the neuropeptide CGRP during pulmonary infections. Thus, blocking the production or activity of CGRP could improve the prognosis of bacterial pneumonia. All these effects can be summed up to the gut/lung microbiota interactions with the immune regulation and with the nervous system, accordingly [100,101]. 

**Figure 6:**
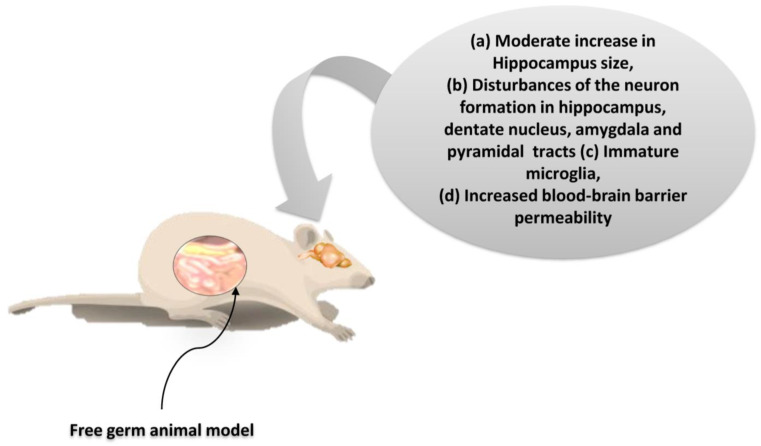
The neurogenesis effects on free germ murine animal model. The restoration of the microbiota in these mice after weaning did not alter neural cell growth. Thus, during the brain developmental periods, gut microbiota regulates the neurogenesis permanently [104,105]. Credits: Original figure by I.A. Charitos.

As suggested by the findings listed above, microbiota dysbiosis is causally related to the consequences not only on the homeostasis of the gastrointestinal tract, but also in other organs. Particularly interesting is the potential link of microbiota with the activities in the CNS. Indeed, many current studies seek the bidirectional correlation of intestinal dysbiosis with higher cognitive functions under intense stress and mental illness (such as depression status) [5,81]. A correlation seems plausible with severe developmental disorders such as autism [41]. Based on these observations, a balanced development of the intestinal microbiota seems to play a crucial role not only in the development of both the intestinal and CN systems, but also in the regulation of their communication [102]. Experimental studies on germ-free (GF) animal models show that the inhibition of colonization by microorganisms strongly influences the expression and function of neuroregulatory substances both in the CNS and ENS, resulting in a dysfunctional gut/brain axis. In the context of this dysfunction, a reduction in the kinetics and sensory activity of the gastrointestinal tract, as well as neuromuscular dysfunction, are observed (Figure 6) [103].

Sometimes, once eubiosis is reached in the gut microbiota, this kind of dysfunction is also solved [106,107]. Results from experimental animal models suggest that the lack of an eubiotic or even absent gut microbiota may alter behavior as well as memory processes by causing hypersensitivity to stressful stimuli. On the same line, other studies on animal models, whose gut microbiota underwent changes by antibiotics or probiotics, demonstrate that the microbiota composition clearly influences the physiology of the CNS, varying the expression of genes that regulate the secretion of certain hormones, neurotransmitters, and neuro-regulators which are responsible for a variety of nervous system processes [106]. Finally, an in vivo study on mice showed that microbiota may be crucial for the management of stress or other stress-related psychogenic disorders [1,107]. One simple mechanism by which the microbiota appears to influence the gut/brain axis is through the modification of the intestinal barrier and the intestinal permeability of the intestinal mucosa [2,106]. The intestinal barrier is the natural barrier between the gastrointestinal tract and the environment. The intestinal barrier performs important functions such as preventing the entry of pathogens, regulating the exchange of useful molecules, and preventing the loss of water and electrolytes while catalytically contributing to the absorption of nutrients [108,109]. The microorganisms that colonize the intestinal mucosa play an essential role in maintaining the intestinal barrier, and its alteration with the use of antibiotics seems to reduce the intestinal barrier protection while its enhancement with probiotics seems to strengthen it. The reduced defense, after deterioration of the intestinal mucosa due to intestinal dysbiosis, leads not only to gastrointestinal dysfunction but also to direct effects on the CNS [2,104] The microbiota influences the gut/brain axis through its effect on the afferent sensory nerves, with subsequent modulation of the higher cognitive functions, the expression of neurotransmitters, the hormonal secretion of the autonomic nervous system, and the absorption of trace elements. Indeed, bacteria from *Lactobacillaceae* phyla that produce nitric acid and hydrogen sulfide also affect homeostasis and trigger nerve signals which regulate bowel motility, discomfort, and pain [92,110,111]. Being the main recipient of the products of bacterial metabolism, the ENS is the site of production of SCFAs such as butyric and acetic acid, whose mediated stimulation of the PNS and serotonin secretion may modify memory and other higher cognitive functions. Therefore, eating habits or taking medications that influence the populations and metabolism of intestinal bacteria can significantly affect both the perception and behavior of the host [41,92,102]. Finally, the ability of intestinal microbiota to influence the availability of nutrients can impair the endocrine function of cortisol- and noradrenaline-producing cells. Since hormones have a fundamental role in regulating mood, perception, reflexes, and the management of stressful stimuli, dietary habits, drug intake, chemicals, and other toxic xenobiotics that can qualitatively and quantitatively affect the gut microbiota composition could significantly influence both hosts’ behavior and perception through the gut/brain axis [95,112,113,114,115,116,117,118]. The intestinal microbiota significantly affects the gut/brain axis and, on the higher functions of the CNS, also affects the endocrine pathways [119]. Another important mechanism through which the microbiota acts on the gut/brain axis is related to the activation of the immune system. In this regard, it is accepted that the use of antibiotics causes inflammatory reactions in the gastrointestinal tract by activating the corresponding immune responses in the inflamed areas of the ENS, and that restoration of the intestinal species of the *Lactobacillaceae* family concomitantly repairs the damage [95,99]. The hypothesis here is that dysbiosis activates the innate immune responses which affect the intestinal–brain axis, with consequential increased epithelial permeability, intestinal motility, and pain. Similar immune responses and effects on the intestinal axis result from the colonization of the gastrointestinal tract by exogenous pathogenic microorganisms such as *Helicobacter pylori* [99,119,120].

### 4.3. The Effect of the CNS on the Gut Microbiota

**Figure 7:**
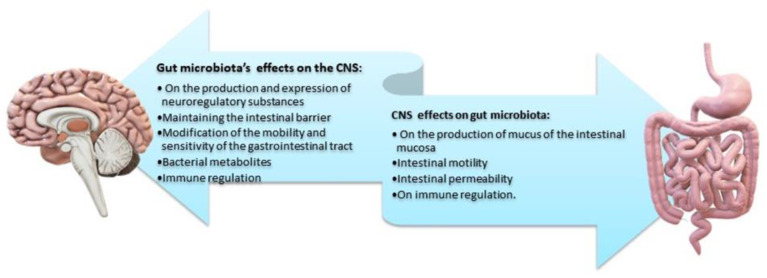
Main effects of the bidirectional interaction between gut microbiota/brain. Microbial metabolites reach the brain and inhibit myelin formation in the prefrontal cortex, preventing the differentiation of Sox10 or MYRF precursor oligodendrocytes. An increased production of metabolites inhibits myelin formation. Decreased myelin is associated with anxiety, depression, and reduced sociability [5,125,126]. Credits: Original figure by I.A. Charitos.

The brain plays a key role in the gastrointestinal tract as it modulates, independently and in parallel with the ENS, the motility of the gastrointestinal tract, the secretion of gastric fluid and mucus, and the related immune responses [121,122]. Given its bidirectional interaction, the gut/brain axis, through stimuli (i.e., stressors), influences the composition and balance of the intestinal microbiota. It has been recurrently observed that short-term stressful stimuli can affect certain microbial portions of the gut microbiota via the afferent neural pathways of the ENS. Dissimilar stressors have different effects on the intestinal mucosa [123] and may modify the secreted mucus both in the quantity and quality of its composition. The long-term stress of daily life is the culprit of intense quality changes in the balance of the intestinal microbiota and subsequent gastrointestinal dysfunction [1,124]. This influence is mediated by the secretion of specific signaling molecules of CNS neurons and by the activation of immune cells. Thus, the effect of the CNS on the composition of the intestinal microbiota largely depends on the presence of specific receptors and neuro-regulatory substances on the surface of the bacteria [1,112,125]. Finally, as already mentioned, continuous stress in the gastrointestinal tract enhances permeability and reduces the protective shield of the intestinal barrier. Greater permeability allows for the entry of pathogenic and bacterial antigens, causing subsequent immune responses that impair, even further, the intestinal microbiota (Figure 7) [2,6].

**Figure 8:**
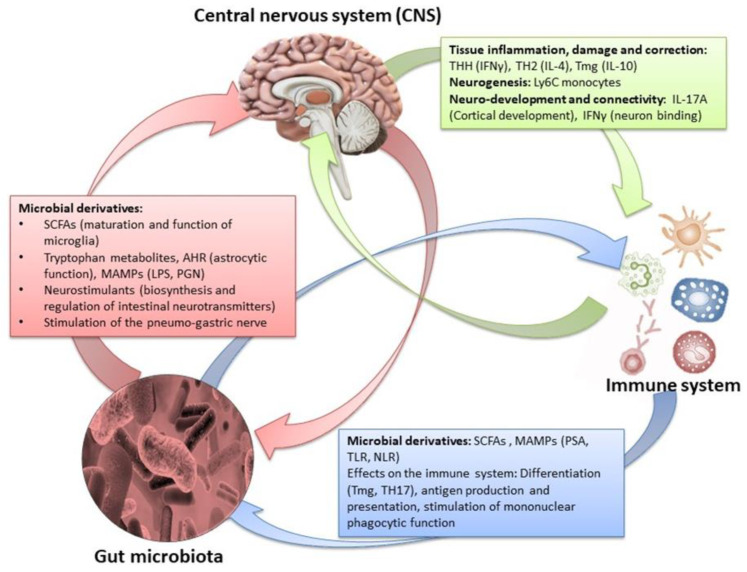
The effects of the amphidromic connection between gut microbiota/brain in relation to the peripheral immune system and metabolome [1,5,43,127,128,129,130,131,132]. Credits: Original figure by I.A. Charitos.

By influencing the processes of synaptogenesis, the intestinal microbiota contributes to the production of various neurotransmitters (such as serotonin), the development of the dopaminergic system, and sometimes the permeability of the blood brain barrier [76,93]. Concomitantly, the products of gut microbes (SCFAs, lipopolysaccharides) can have more explicit effects in specific areas of the brain. Through the hypothalamic–pituitary–adrenal axis, the CNS-induced changes in intestinal permeability, secretory activity, and gastrointestinal motility (and through the ANS) may modify the microbiota, which, in turn, can alter immune processes [2,41]. Finally, changes in endocrine mediators and related neuroregulatory substances may reflect the CNS response to extrinsic stimuli (Figure 8) [5,126].

## 5. Gut/Brain Axis and Diseases

### 5.1. Neurological Disorders

Multiple sclerosis (MS) is a chronic autoimmune neurodegenerative disease whose main feature is the gradual demyelination of nerve cells in the CNS. Myelin is the protective layer of axons. The autoimmune response leads to even more negative results due to the reduced immunosuppression by Tregs cells in individuals with multiple sclerosis. An abnormal immune response via CD4 T lymphocytes and the secretion of proinflammatory cytokines by the hyperactivation of Th1 and Th17 cells will lead to the penetration of immune cells into the CNS, activating an immunosuppression of the surrounding neurons [133]. In sclerosis, damaged myelin is replaced by plaques of scar tissue, and this process is described as demyelination [134]. Various hereditary, infectious, and environmental factors have been implicated as causative factors. Viral infection by HBV (Epstein–Barr Virus) or HSV-6 (Herpes Simplex Virus 6) are among the possible widely studied causes, while other studies are considering the possible role of the gut microbiota in triggering autoimmunity [135,136]. The gut microbiota is thought to play an important role because it can control immune signals which are activated under multiple sclerosis. Indeed, studies in animal models with CD4+ T-induced autoimmunity have shown that the administration of antibiotics leads to a reduction in the severity of the disease symptoms as well as a greater differentiation and accumulation of Foxp3+ Tregs [137,138,139]. Recent investigations of the gut microbiota in patients with multiple sclerosis have observed changes in microorganism composition over the course of the disease. A progressive decrease in 21 microbial species from the phyla *Bacillota* and *Bacteroidetes* were concomitantly accompanied by an increase in the populations of the archaea *Methanobrevibactacteriaceae*. It has been noted that a reduction in *Clostridia* spp. and *B. fragilis* causes insufficient induction of T lymphocytes that exhibit immunosuppressive-immunoregulatory activity in the large intestine of patients with multiple sclerosis [140,141]. Also, there is an association between specific strains of the microbiota with immune markers such as IL-17 [142]. The archaea are anaerobic germs whose lipid membranes and cell wall are highly immunogenic, which is consistent with an induction of local or systemic inflammation, as in the case of multiple sclerosis. Instead, populations of microbials with anti-inflammatory properties such as the phyla bacteria *Bacteroidota* and *Bacillota* are reduced. Interestingly, these population changes appear to normalize after antibiotic therapeutic use [143,144]. Other investigations underline significant differences in the classes of bacteria which compose the microbiota in patients with multiple sclerosis (not receiving therapeutic treatments) compared to normal individuals; for example, bacteria such as *Akkermansia muciniphila* and *Acinetobacter calcoaceticus* have been detected in multiple sclerosis patients, but not in normal individuals. Furthermore, it is hypothesized that the transport of bacteria of these species into muscles (that do not have a microbiota) has caused autoimmunity [140,141]. These observations suggest that changes in inflammatory or anti-inflammatory epigenetic factors in the gut microbiota composition of multiple sclerosis patients may contribute to the pathogenesis of the disease. Similar studies in patients before and after treatment have identified changes in the populations of many microorganisms of different genera. These correlations are studied at the level of alteration of specific inflammatory factors and at the level of immune responses through specific immunogenic biomolecules and immunoregulatory mechanisms [144,145]. Finally, similar observations have been documented for other demyelinating autoimmune diseases such as Guillain-Barré syndrome (which causes gradual demyelination of the PNS) and Devic’s disease, characterized by extensive demyelination of the optic nerve [71]. Despite the specific processes by which intestinal microbiota can trigger the onset or influence the course of an autoimmune demyelinating disease, they have not been clearly identified so far, although several hypotheses have been proposed [145]. These are generally shared between all autoimmune diseases, and include molecular mimicry, bystander activation, and the presence of a persistent infection with—or even without—local microbial spread (epitope spread). Since the quality and quantity of the microbiota composition depends on and reflects the quality of the diet, specific habits, and other environmental factors which are characteristic of the modern lifestyle, it is likely that the study of the gut microbiota may provide some hints for the increased epidemiology of these diseases [146,147]. Accordingly, alterations in bile acid metabolism with lower levels of primary and secondary bile acids, and a dysregulation in tryptophan metabolism, were observed in Parkinson’ patients and were associated with an increased risk and clinical severity of the disease [148].

Alzheimer’s disease is a progressive neurodegenerative syndrome which affects the patients mental and motor functions. The main feature of the disease is the irrregular conduction of nerve impulses in those areas of the CNS where β-amyloid plaques form and accumulate, leading to an abnormal structure of brain tissue proteins. Studies using metabolomics/lipidomics have consistently reported alterations in several metabolic pathways, such as those involved in mitochondrial bioenergetics, methionine, arginine, glutamate, lipids, and fatty acid biosynthesis. The accumulation of hyperphosphorylated tau protein also occurs within cortical neurons [149,150,151,152,153,154]. The infectious origin of this disease has been associated with contaminations by bacteria (such as *Spirochetes* and *Chlamydophila pneumoniae*), viruses (such as HSV1) and fungi. The correlation with infection from HSV1 has been related to the increased expression of genes which encode for cholesterol hydroxylase, which is in turn associated with the overproduction of β-amyloid [155,156,157]. It should be mentioned that the intestinal microbes produce β-amyloid and large amounts of lipopolysaccharides (LPS), whose pro-inflammatory activity may contribute to the physiology of the disease. In conditions of intestinal dysbiosis, the impaired intestinal barrier facilitates the passage of cytotoxic substances, and the LPS further strengthens the production of inflammatory cytokines [41,158,159,160]. Furthermore, structural errors of extracellular amyloid proteins can cause their recognition as PAMPS (pathogen-associated molecular patterns), which trigger inflammation through stimulation of the TLR2 receptor [161]. In addition, some bacteria phyla such as *Bacillota*, *Bacteroidota*, and *Pseudomonadota* produce some exogenous amyloid proteins which may have errors in their quaternary structure. This, in turn, can trigger immune responses even against endogenous β-amyloids, accelerating the progression of the disease. On the other hand, the microbiota converts the indigestible polyphenols, introduced with the diet, into phenolic acids (3-hydroxybenzoic acid, 3- (3’-hydroxyphenyl) and propionic acid, whose beneficial effects have been reported [162]. It was noted that, in *Mus musculus* models, characterized by the absence of gut microbiota and the concomitant hyperaccumulation of β-amyloid, the transferring of gut microbiota from healthy mice helped to improve their health by reducing the accumulation of β-amyloid. The fecal microbiota from mice with Alzheimer’s disease and healthy mice showed a significant increase in *Verrucomicrobia* and *Pseudomonadota* phyla in those mice that developed the disease, while the population of the genus *Ruminococcus* and *Butyricicoccus* showed a clear reduction. Thus, some microbial species of the intestinal microbiota activate molecular pathways which are associated with β-amyloid and may have their role in the pathogenesis of Alzheimer’s disease [41,163,164,165]. 

Parkinson’s disease is another chronic degenerative disease of the CNS, whose pathogenesis results from a selective degeneration of some dopamine-producing neurons. Dopamine is a key neuroregulatory substance, the lack of which causes severe motor impairment [166,167]. The disease is characterized by the loss of dopaminergic receptors in the substantia nigra, accompanied by the accumulation of α-synuclein and Lewy particles in the remaining neurons. In a study on lipid and purine metabolism, it was noted that compounds with medium and long chain fatty acids (5-dodecanoate, 3-hydroxydecanoate, docosadienoate, and docosatrienoate), lysolipid 1-arachidoylglycerophosphocholine, and several purine compounds may retain a predictive importance regarding disease progression [168]. In more recent years, efforts to investigate potential regional factors associated with the disease have observed that α-synuclein accumulation, before appearing in the CNS, begins in the intestinal nervous system, and that it is associated with some gastrointestinal symptoms. Once again, the hypothesis is that the degenerative disease may start from the intestine, and, precisely, from a dysbiosis of its microbiota [169,170,171]. In support of this concept, the intestinal microbiota of Parkinson’s patients shows significant differences with respect to healthy individuals [172,173,174,175]. Major changes include drastic reduction in populations of *Bacteroidetes* phyla and *Prevotellaceae* spp., but also reduced SCFAs, which, as mentioned, are important catabolites of complex carbohydrates by intestinal bacteria [176]. As for other conditions, metabolites of the gut microbiota may significantly impact the host’s defence system, representing a potential link for the correlation between changes in the microbiota and the development of Parkinson’s disease. The SCFA levels might act as biomarkers, as demonstrated by the beneficial effects on patients treated with antibiotics and probiotics. It has been noted that *Enterobacteriaceae* family populations tend to increase, and the degree of their growth appears to be related to the degree of development of disease-specific symptoms. Although a link between changes in microbiota composition and disease progression has been repeatedly suggested, the significant variation in outcomes among patients requires further investigation [177,178]. Interestingly, when mice sterile intestine was colonized with microbiota from patients with Parkinson’s disease, the pathological organic dysfunctions were much more evident than those observed in mice colonized with the microbiota of healthy subjects. Furthermore, an increase in pro-inflammatory bacterial populations with a concomitant reduced number of anti-inflammatory bacteria was noted in fecal content and biopsies at the sigma intestinal tract [179]. In addition, the gut microbiota alteration in populations of bacteria from the genus *Enterobacteriaceae* and the genus *Lachnospiraceae* has been associated with the disease severity regarding motor and non-motor impairment [178,180,181].

Chronic fatigue syndrome (CFS) and myalgic encephalomyelitis (ME) are characterized by a chronic feeling of fatigue with no other pathological causes, and are usually accompanied by headache, insomnia, muscle aches, and a general feeling of malaise. The etiology is still unclear but appears to be related to various infectious agents in combination with disorders of immune and hormonal responses as well as psychological disturbances [182,183]. Metabolite levels, changes in which have been related to clinical severity, include those derived from the cholesterol/bile acid synthesis, branch chain amino acid metabolic intermediates, products of the lanosterol pathway, sphingolipids, glycosphingolipids, purines, microbiota’s aromatic amino acid metabolites, flavin adenine dinucleotide (FAD), and an increase in pyrroline-5-carboxylate and arginine [184]. These patients exhibit chronic lymphocytic hyperactivity and cytokine overexpression, which appear to exacerbate pathogenesis. Many recent studies have investigated symptoms in relation to changes in gut microbiota composition, as increased immune and inflammatory factors may be ascribed to gut dysbiosis [38,185]. The biodiversity of the intestinal microbiota in CFS patients appears generally decreased, with a reduction in the genus *Bifidobacteria* (phylum *Actinomycetota*) and *Bacillota* phylum populations and an increase in *Enterococcus* spp. and *Streptococcus* spp. [177]. Indeed, the concomitant morbidity between myalgic encephalomyelitis/chronic fatigue syndrome (ME/CFS) and several gastrointestinal syndromes has been reported. Furthermore, approximately 92% of patients with this syndrome may develop irritable bowel syndrome [186]. With respect to healthy individuals, other studies have shown an increase in the mucus production in the intestinal tract and elevated levels of proinflammatory cytokines such as IL-6, IL-8, IL-1β, and TNFα in patients with both syndromes, concomitantly accompanied by changes in the microbiota composition [177,187]. Finally, several bacteria associated with the butyric acid production (such as the *Ruminococcaceae* family) do not appear to be present or are present only in limited amounts (such as *Bacteroides* genus) in the intestinal tracts of individuals with irritable bowel syndrome and ME/CSF [188,189,190,191]. Findings are not always consistent. However, although the role of the intestinal microbiota in the activation or development of the disease requires additional investigation, clinical studies show that therapeutic interventions aiming to correct intestinal dysbiosis may ameliorate the symptoms of chronic fatigue [192].

### 5.2. Neuro-Psychopathological Diseases

As we mentioned previously, intestinal microbiota contributes to the secretion of neuroregulatory substances from the CNS, such as dopamine, serotonin, and melatonin, which are profoundly involved in mood and mental function. Thus, the microbiota can play an important role in the management of mood disorders such as depression or anxiety [76,193]. 

Depression (or major depressive disorder) is characterized by a feeling of intense and prolonged sadness and can be triggered by various reasons (such as a sad event, a loss, or a post-traumatic condition) but its intensity and duration are disproportionately greater than the event. It is a neuropsychiatric disorder with features of immune deregulation [88,194]. In many studies, it has been reported that the tryptophan, tyrosine, and purine derivatives are differently expressed in patients with major depression, suggesting that metabolic components in the kynurenine pathway are plausible mechanisms concurring to the disease’s pathophysiology [195]. The microbiota of these patients have specific differences compared to healthy individuals. Patients with depressive disorder appear to have increased concentrations of the phyla *Actinomycetota*, *Pseudomonadota* and *Bacteroidota*, and reduced levels of *Bacillota* phylum and *Lactobacillaceae* phylum populations. Indeed, species from the *Lactobacillaceae* family have been found to have antidepressant and anti-inflammatory effects. Furthermore, the increased presence of bacteria of the genera *Enterobacteriaceae* and *Allistipes*, and the reduced appearance of *Faecalibacterium* genus, have been associated with the severity of depressive symptoms [1,41,91,94,196]. No differences were observed between the microbiota of depressed women and men (showing that the differences were related to the disorder and not to sex/gender) [197]. However, as for several other conditions described in this manuscript, results are not always consistent, and are sometimes conflicting. In part, these differences may be explained by the natural gut microbiota diversity from person to person during their lifetime. One of the main mechanisms by which the microbiota is implicated in the development of mood disorders is the weaker intestinal barrier, a feature of intestinal dysbiosis [1,104]. Hence, individuals with depressive symptoms often exhibit an increased expression of pro-inflammatory cytokines, such as -1β, IL-6, and TNF-α, as well as interferon gamma and C-reactive protein levels [198,199]. The gut microbiota influences the transcription of these same cytokines, with dysbiosis triggering the so-called inflammatory pathway, while beneficial metabolites (once again, SCFAs) limit the production of pro-inflammatory cytokines, such as Interleukin (IL)-1 [200,201]. The gut microbiota is known to support the integrity of the tight junctions between enterocytes. More and more studies support the existence of an inflammatory component in depression. Indeed, anti-inflammatory drugs, such as COX-2 inhibitors, have previously shown efficacy in major depression [202]. The reduced functionality of the intestinal barrier allows for the absorption of bacteria and endotoxins that trigger a chain of immune responses, resulting in the increased expression of inflammatory cytokines with a direct effect on the function of neurotransmitters, and thus influencing the mood in the CNS [1,5]. Importantly, this is an amphidromic mechanism, in which a bad mood can exacerbate intestinal dysbiosis through the gut–brain axis, creating a vicious circle that changes the gut microbiota composition and further worsens the condition; for this reason, analysis of the gut microbiota has been proposed as a useful biomarker in the management of depression, but further clinical trials are needed [5,153]. Finally, the bacterial LPSs, mainly derived from the *Enterobacter* spp., may play a crucial role in major depression, and its levels are known to be higher in patients with severe depression than in healthy individuals. This metabolite enters the systemic circulation through permeability defects of the intestinal narrow epithelial junction, thus creating leaky gut syndrome and, subsequently, antibodies against LPS are produced which can further destabilize the axis of the gut/brain microbiota [41,172,203,204].

**Figure 9:**
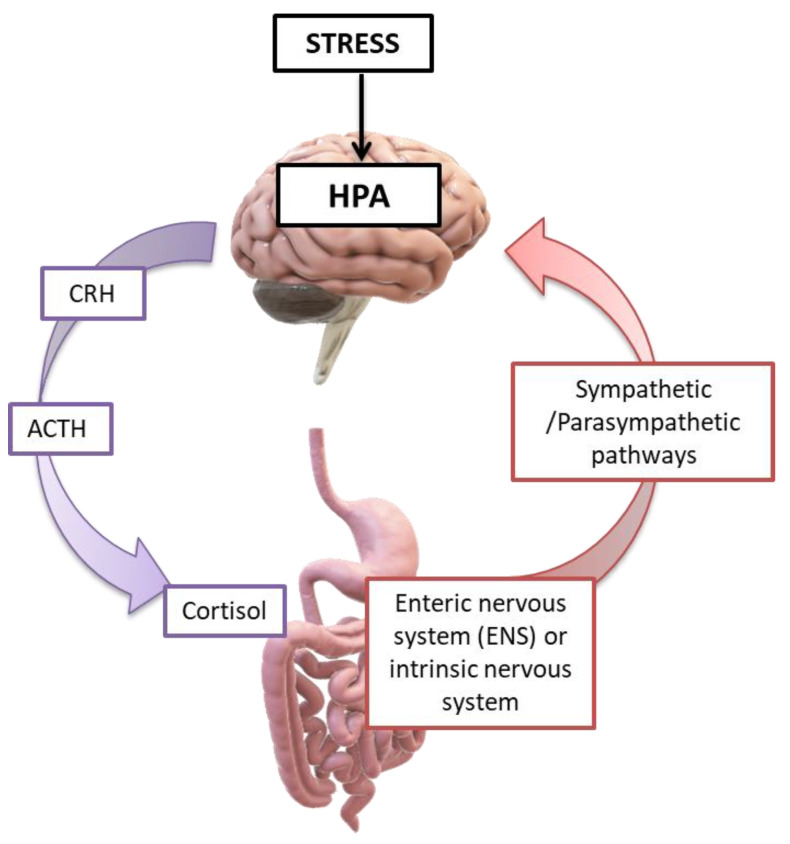
Intestine/brain axis in stressful environmental conditions: stress activates the hypothalamic–pituitary–adrenal axis (HPA axis), leading to the hypothalamus secretion of the corticotropin releasing hormone (CRH) and, subsequently, the secretion of the adrenocorticotropic hormone (ACTH) from the pituitary gland. This, in turn, leads to the secretion of cortisol by the adrenal glands. Cortisol acts in the CNS communication pathways, hormonal and neural, which, interacting, influence the activities of the cells: intestinal effector, smooth muscle, epithelial, enterochromaffin, interstitial Cajal’s cells, enteric neurons, and immune cells. Thus, stress conditions cause the variation in microbiome, immune function, mucus, intestinal motility, and permeability [1,5,203]. Credits: Original figure by I.A. Charitos.

Anxiety disorders are a set of mental illnesses characterized by greater sensitivity to stressful stimuli (anxiety, fear, or panic) without being able to justify these feelings. Such disorders include generalized anxiety disorder, panic attacks, post-traumatic stress disorder, and various types of phobias (such as agoraphobia) [205]. In recent years, research has largely focused on the association of the pathophysiology of “stress” in relation to the gastrointestinal tract and, therefore, to its microbiota. Stressful environmental and psychosocial factors have significant effects on the functioning of the gastrointestinal tract and the immune system; therefore, affecting the microbiota differently in a short-term or prolonged/chronic way [1,41]. Indeed, an episode of excessive anxiety or fear can cause indigestion or gastric pain, while prolonged stress in the context of an anxiety disorder has also been blamed for severe gastrointestinal disorders such as esophageal reflux, gastric ulcers, inflammatory bowel syndrome (such as ulcerative colitis), and more [81,105]. Hence, the main effects of stress on the gastrointestinal tract via the intestinal/brain axis include changes in intestinal motility, visceral discomfort, altered secretory capacity, and permeability of the intestinal mucosa. These changes significantly affect the composition and function of the gut microbiota, causing a possible state of dysbiosis, which in turn contributes to the worsening of anxiety symptoms in the context of the gut/brain axis [2,41]. Thus, there may be possible underlying immune and neuroregulatory mechanisms that mediate the effects of stress on the gastrointestinal tract (Figure 9) [105,106,169]. 

On top of contributing to the functionality of the intestinal barrier, the integrity of the tight junctions, and the immune regulation, the intestinal microbiota has a primary impact on the modulation of intrinsic primary afferent neurons, and the production of bacterial metabolites concur to the activation of neurotransmitters (GABA, Serotonin, Tryptophan (5-HT), and others) and neurotrophic factor (BDNF) [76,96,121]. The fecal microbiota of patients with generalized anxiety disorder showed a reduced composition in the genera *Faecalibacterium*, *Eubacterium*, *Lachnospira*, *Butyricicoccus*, and *Sutterella*, suggesting a potential role of these genera in the maintenance of mental health through the production of SCFAs [206,207].

Autism is one of the most controversial associations with the gut microbiota and brain axis. Autism is a neuro-psychological disorder which does not belong to psychiatric diseases but to the category of Pervasive Developmental Disorders (PDDs), now called autism spectrum disorder, that occurs in early childhood (2–3 years) [68,208]. It is characterized by reduced interaction with the environment, such as limited development of communication, social, and cognitive skills. The severity of the symptoms is widely variable and ranges from mild forms with minor problems in the development of normal psychosocial functions to forms of high morbidity and absolute disability to social communication and interaction [172]. Children with autism frequently suffer from persistent gastrointestinal disorders that often vary with the severity of autism symptoms [136,137]. In fact, they show a reduced biodiversity of the microbiota with a characteristic increase in some *Clostridium* genera, *Bacteroidota*, *Bacillota* phyla, *Bifidobacterium*, and genera from the *Lactobacillaceae* family [209,210]. They also have some elevated SCFAs (such as propionic acid) in their gastrointestinal tract, a clear sign of intestinal dysbiosis which can affect the expression of certain genes associated with CNS development. Thus, SCFAs are thought to be involved in the development of autism spectrum disorders [9]. Hence, SCFAs regulate the release of intestinal peptides from enteroendocrine cells and have been shown to regulate the synthesis of gut-derived serotonin (producing 95% of total serotonin) by enterochromaffin cells of serotonin, most of which are present in plasma. Both, in turn, affect gut–brain hormonal communication [130]. Besides playing a role in both peripheral metabolism and intrinsic functions in the gastrointestinal tract, serotonin can locally activate afferent nerve endings which are directly connected to the CNS [211]. Indeed, elevated plasma serotonin has been observed in children with autism [212,213]. Furthermore, an inverse link between high plasma serotonin and low serotonergic neurotransmission has been demonstrated in young adult males with autism spectrum disorder [211]. Given the extensive range of autism clinical manifestations together with the wide diversity of the gut microbiota in patients, the research investigating a potential causal relationship between the gut microbiota and autistic disorders is still in the process of further exploration [214,215]. 

Psychogenic eating disorders are characterized by extreme eating habits that can lead to severe malnutrition or overnutrition (such as anorexia nervosa and bulimia nervosa) with various degrees of morbidity. Bulimia is characterized by compulsive or overeating that can be occasional or permanent and lead to obesity [216]. Although eating disorders are classified as psychogenic and their exact etiology is unknown, current studies are also studying the biological factors involved in their pathology. Given the well-established association of intestinal dysbiosis with mental processes, there is a strong research interest regarding the role of the gut microbiota and gut–brain axis mediation in their pathogenesis, although the current literature is still limited [217]. A possible mechanism by which the gut/brain axis may be involved in the development of eating disorders is the effect of the microbiota on the production and modulation of specific hormones that regulate appetite [218]. Another possible mechanism involves the production of peptides by the gut microbiota which, similar to hormones, regulate appetite, causing abnormal immune processes that deregulate the sensation of appetite. Therefore, in this case, the intestinal–cerebral axis can cause an eating disorder whose pathology exacerbates intestinal dysbiosis by causing a vicious circle [21,41,219]. 

## 6. The Modulation of the Gut-Brain Axis via Probiotics, Psychobiotics, and Prebiotics

Probiotic products and their benefits were already known from ancient times as a treatment for certain diseases or even poisonings [35,220]. Probiotics are products that essentially contain beneficial living microorganisms that normally colonize the intestines. The purpose of the consumption of probiotics is the enrichment of the intestinal microbiota with “friendly” strains of microorganisms whose populations are insufficient due to an unbalanced diet, drugs (such as antibiotics), intoxicants, or pathological reasons (such as oral cavity infections, urogenital disorders, bulimia nervosa, and others) [221,222,223,224,225,226]. The most common probiotic products are dairy products (such as Greek yogurt) and contain lactic bacteria (such as those from the *Lactobacillaceae* family) and *Bifidobacterium* spp. *Lacticaseibacillus casei* is considered an immune enhancer while *Bifidobacterium animalis* regulates intestinal motility by accelerating the passage of food, the immune response, and others [98,227,228]. Recently, the oral administration of probiotics has been repeatedly proposed to ameliorate clinical conditions of SARS-CoV-2 patients. The oral intake of *Lactobacilli* spp. (such as *L. casei* ATCC 39392, *Lactobacillus delbrueckii* subsp. *bulgaricus* OLL1073R-1, *Lactiplantibacillus plantarum* subsp. *plantarum*, and others), and/or *Bifidobacterium* spp. (such as *B. lactis* DSM 32246B, *B*. *lactis* DSM 32247, *B. short*, and others) is regarded as an adjuvant therapy which reinforces the immune defenses and alleviates some symptoms, therefore helping to improve the clinical outcome [6,82,229]. Thus, modification of the intestinal microbiota through diet or the use of probiotics, antibiotics, and other therapeutic interventions is regarded as a conceivable tool in the personalized treatment of mood disorders and other psychiatric diseases [51,66,224,230]. Particularly vulnerable to antibiotics are populations of the *Lactobacillaceae* family and *Bifidobacterium* spp., and, thus, the increased consumption of antibiotics by infants or children can lead to early dysbiosis effects which are not only limited to the intestinal microbiota, but which also affect the entire microbiota of the organism. Therefore, in the context of a correct use of antibiotics, it is necessary to consider their effect on the intestinal microbiota, which can be drastically reduced even after short antibiotic treatments [172,223]. Several studies have demonstrated the beneficial effect of probiotics on mental mood and psychopathological diseases. Taking probiotic supplements has been observed to have a positive effect on anxiety disorders, chronic fatigue syndrome, and depression. Species from the *Lactobacillaceae* family and *Bifidobacteria* spp. populations have beneficial effects in patients with depressive disorder [229,230,231,232]. *Lactobacilli* spp. have been found to affect the levels of neurotransmitters and hormones such as corticosterone, helping to relieve symptoms of anxiety and depression [233]. Furthermore, a few studies indicate the possible beneficial effect of probiotics in restoring intestinal dysbiosis and autism symptoms [5,231]. The advantageous effects of probiotics on anxiety disorders reinforce the concept of the involvement and influence of the microbiota on their occurrence [232]. *L. rhamnosus* may improve the anxiety symptoms of people with depressive behaviors. The probiotic *B. longum* has a similar effect and *Bifidobacterium infantis* has been proven effective in “relieving” the depression and anxiety associated with IBS, likely via increased levels of 5-hydroxytryptophan (5 -HT) made from tryptophan [6,117,232,233,234,235]. Furthermore, the consumption of probiotic milk for about three weeks has significantly improved the psychological profile in subjects. These living organisms are today defined “psychobiotics” [232]. The “psychobiotic” is a live organism which, in adequate quantities, produces beneficial effects in patients suffering from psychiatric diseases, and it has been proposed as an adjuvant treatment in depression. Other psychobiotics are those which can produce norepinephrine, including *Escherichia*, *Bacillus*, and *Saccharomyces*, those able to improve 5-HT such as *Candida*, *Streptococcus*, *Escherichia*, and *Enterococcus*, and, finally, dopamine producers such as *Bacillus* and *Serratia* [236,237].

Prebiotics do not contain live microorganisms but are instead indigestible food components that promote the growth of beneficial symbiotic microbes in the intestinal microbiota. Indeed, the treatment of multiple sclerosis patients with prebiotics enhanced the microbial environment of the intestine and inhibited the inflammation associated with monocytes [238,239]. Furthermore, fecal transplantation from healthy donors has shown its efficiency in dysbiosis, and encouraging results have been documented in various pathologies, including in patients with multiple sclerosis [240,241,242]. Prebiotics create an environment in the intestinal tract that promotes the growth of beneficial microbiota without stimulating the growth of harmful pathogens. Prebiotic components, such as inulin and fructo-oligosaccharides, strengthen the intestinal barrier to create an environment that is suitable for beneficial bacteria but also to prevent the passage of pathogens, and are believed to have anti-cancer effects [243,244]. Therefore, the combined intake of probiotics–prebiotics enhances the composition of the intestinal microbiota and leads to the strengthening of the intestinal barrier and of the intestinal epithelium, a reduction in pathogens or harmful metabolic derivatives, the increased production of antibodies, and a competitive action against exogenous pathogens [5,40,245]. Consequently, probiotics–prebiotics can contribute significantly to the management of a variety of gastrointestinal and metabolic diseases, and also to some neurological and neuro-psychopathological disorders [1,41,233,246,247,248].

## 7. Conclusions

The aim of this work was to highlight the crucial role of the gut–brain axis in relation to the maintenance of good health via microbiota and their metabolites. All of the possible bio-mechanisms of interactions described here may help our comprehension and widen the range of potential targets for the development of more effective treatment strategies. The study of the gut/brain axis remains a very broad field to be explored in biomedical research. The role of the gut microbiota does not stop only in gastrointestinal, metabolic, and immune diseases, but extends its potential influence to some neurological diseases and mental disorders. Since the causes for many of these disorders have not been definitely identified, changes in the intestinal microbiota can be currently regarded either as a causal factor or as a symptom. According to the literature, the bidirectional interaction of the gut/brain axis appears to have a significant effect on all levels of gastrointestinal function, and may extend to several CNS-mediated activities. Despite the growing interest in this field, further and more focused research is clearly needed to identify the exact interaction mechanisms between the microbiota, ENS, and CNS to gain a full understanding of the pathophysiology of many functional and mental organic disorders. In this complex relationship, the most recent evidence suggests that the quantitative and qualitative changes in microbiota composition are paralleled by the importance of specific “good” and “bad” metabolites. In this regard, advances in intestinal microbiota management techniques may allow for positive results, but more studies are required to achieve relevant systematic clinical applications. Nutrition has been highlighted as a special determinant for maintaining a healthy intestinal microbiota and general physical and mental health. The use of probiotics and prebiotics highlights their important contribution as adjuvants not only in therapy but also in fecal transplantation. Understanding the factors that alter the balance of intestinal microbiota (dysbiosis) and the effects of these changes on human physiology is a rich source of useful information that can be beneficial in a variety of applications to treat many diseases. It should be clear that the intestinal microbiota, rather than being a simple metabolic and defense organ, is a complex system of the secretion of molecules which are able to modulate the CNS activities from both a functional and emotional point of view.

## Figures and Tables

**Table 1:** The effects of the microbiota on the brain occur via the nerve pathways to the CNS through the stimulation by the production of neurotransmitters, hormones, and metabolites, with action in the CNS by some bacterial strains. This influences the behavior, the mood, and the attention state of the guest. Many stress conditions have been shown to increase the rate of expression of several neurotransmitters, such as norepinephrine, which in turn makes *E. coli* and *C. Jejuni* strains even more infectious. The main changes caused by stress in the gastrointestinal system are changes in intestinal motility, increased visceral motility, changes in gastrointestinal secretions, increased intestinal permeability, and decreased mucosal regeneration and perfusion capacity. Furthermore, stress can change the composition of the intestinal microbiota, leading to dysbiosis, negatively influencing the populations of beneficial bacteria such as those from the *Lactobacillaceae* family and *Bifidobacterium* spp., and positively affect the growth of potential pathogens. Emotional stress increases the rate of *Lactobacillus* spp. excretion for up to six days after the episode, and this reduction can allow exogenous bacteria to colonize the intestinal epithelium. Thus, we have evidence of the crosstalk gut/behavior axis through the gut/brain axis that modulates the behavioral responses [92,93].

Species/Phyla	Neurotransmitters
*Bacillus*, *Escherichia*, *Saccharomyces*	Serotonin, Noradrenaline [76,94]
*Candida*, *Bacillus*, *Escherichia* spp., *Enterococcus*, *Streptococcus*	Dopamine, Noradrenaline, Serotonin [95]
*Lactobacillaceae*	Acetylcholine [96]
*Bifidobacterium infantis*	Tryptophan (5-HT) [97]
*Bifidobacterium*, *Lactobacillaceae*	γ-aminobutyrate (GABA) [98,99]

## Data Availability

All relevant data are reported in the manuscript.
